# Cardiac manifestations of PRKAG2 mutation

**DOI:** 10.1186/s12881-017-0512-6

**Published:** 2018-01-03

**Authors:** Pooya Banankhah, Gregory A. Fishbein, Anthony Dota, Reza Ardehali

**Affiliations:** 1 Department of Internal Medicine, Olive View-UCLA Medical Center, 14445 Olive View Dr., Sylmar, CA 91342 USA; 2 Division of Anatomic Pathology, Department of Pathology and Laboratory Medicine, David Geffen School of Medicine at UCLA, 10833 Le Conte Ave, CHS 1P-326, Los Angeles, CA 90095 USA; 3 Heart Center of Nevada, 700 Shadow Ln Ste 240, Las Vegas, NV 89106 USA; 4 Division of Cardiology, Department of Internal Medicine, David Geffen School of Medicine at UCLA, 675 Charles E Young Dr. S. Room 3760, Los Angeles, CA 90095 USA

**Keywords:** Cardiovascular pathology, Hypertrophic cardiomyopathy, PRKAG2 cardiac syndrome, Wolff-Parkinson-white syndrome

## Abstract

**Background:**

The Protein Kinase AMP-Activated Non-Catalytic Subunit Gamma 2 (PRKAG2) cardiac syndrome is characterized by glycogen accumulation in the cardiac tissue. The disease presents clinically with hypertrophic cardiomyopathy (HCM), and it is often associated with conduction abnormalities.

**Case presentation:**

A 23 year-old female with history of Wolff-Parkinson-White (WPW) and HCM presented for evaluation after an episode of Non-ST Elevation Myocardial Infarction (NSTEMI). The patient was found to have severe coronary bridging on angiography and underwent an unroofing of the left anterior descending artery (LAD). Due to the constellation of symptoms, the patient underwent genetic testing and a cardiac muscle biopsy. Genetic testing was significant for an Arg302Gln mutation in the PRKAG2 gene. Cardiac tissue biopsy revealed significant myocyte hypertrophy and large vacuoles with glycogen stores.

**Conclusion:**

The pathologic and genetics findings of our patient are consistent with PRKAG2 syndrome. Patients presenting with conduction abnormalities and suspected HCM should be considered for genetic testing to identify possible underlying genetic etiologies.

## Background

HCM is a heterogeneous cardiovascular disease with the prevalence of 1 in 500 in the general population [1]. Clinical diagnosis of HCM requires a hypertrophied, non-dilated left ventricle without evidence of any other cardiac or systemic disease that could produce the observed hypertrophy [1]. Sarcomere protein gene mutations have been associated with up to 60% of familial or sporadic cases of HCM [2]. However, recent studies have led to identification of non-sarcomeric causes, including inborn errors of metabolism, neuromuscular diseases, mitochondrial diseases, malformation syndromes, and drug reactions that mimic HCM [2]. PRKAG2 cardiac syndrome is an autosomal dominant metabolic heart disease characterized by left ventricular hypertrophy (LVH), progressive conduction abnormalities, and ventricular pre-excitation. The prevalence of PRKAG2 syndrome is 0.23–1% in patients with suspected HCM [3].

Cardiomyopathies caused by glycogen storage diseases including PRKAG2 mutations are distinguished from other types of HCM by the formation of glycogen filled vacuoles in myocytes [4]. The glycogen accumulation is often associated with an eccentric pattern of hypertrophy and conduction abnormalities that characterize the PRKAG2 syndrome. This case report describes the clinical presentation and pathologic findings of a patient with a PRKAG2 mutation who presented with angina and palpitations.

## Case presentation

A 23 year-old female with history of WPW, HCM, and pseudotumor cerebri was referred to our center for further evaluation and treatment of persistent angina. The patient had a history of WPW syndrome, for which a catheter ablation had previously been performed. The patient had been experiencing frequent angina both at rest and with exertion. During hospitalization at the referring facility for a ventriculoperitoneal shunt repair, the patient suffered a NSTEMI and was immediately transferred to our center.

On admission, electrocardiogram showed normal sinus rhythm with left atrial enlargement, left ventricular hypertrophy with repolarization abnormalities, and an incomplete right bundle branch block. The patient’s echocardiogram revealed a dynamic left ventricle with an ejection fraction of 70–75% and end-diastolic septal thickness of 2.2 cm without presence of systolic anterior motion of mitral valve. A cardiac magnetic resonance imaging study confirmed prominent hypertrophy in the apical and septal regions. Given her persistent angina, a coronary angiogram was performed that revealed severe coronary bridging with compression of the proximal to mid LAD with near complete compression of all the septal perforator branches. In addition, the left ventricle was noted to be spade-shaped with substantial hypertrophy in mid to apical regions.

The presence of HCM in the setting of previous WPW syndrome raised suspicion for an underlying genetic disorder. In addition, patient reported a family history of an uncle with WPW. Genetic testing using Illumina next generation sequence analysis revealed a missense Arg302Gln mutation in the PRKAG2 gene, confirming the clinical diagnosis of glycogen storage cardiomyopathy. This missense heterozygous mutation on exon 7 replaces arginine with glutamine at codon 302 of the PRKAG2 gene. The PRKAG2 gene consists of 560 amino acids with a molecular mass of approximately 63kd [5]. The patient was also found to have a heterozygous CACNB2 mutation on exon 4, which is of unclear clinical significance.

The patient underwent an unroofing of the LAD and a radical extended myomectomy of septal muscle through a left ventricular apical approach. The atrial and ventricular muscles were noted to have significantly increased thickness during the surgery, which was previously observed on the echocardiogram. The patient was also found to have an abnormal papillary muscle attached to the anterior mitral leaflet, which was surgically resected. Post-surgical transesophageal echo showed a pseudo normal diastolic left ventricular filling. There were no post-operative complications. At 3-month follow up, the patient remained symptom-free with significantly improved exercise tolerance.

**Fig. 1:**
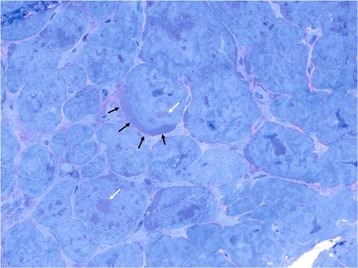
1 μm thick section stained with Toluidine blue. Arrows indicate intracellular glycogen accumulation both in vacuoles (white) and sub-sarcolemmal (black)

**Fig. 2:**
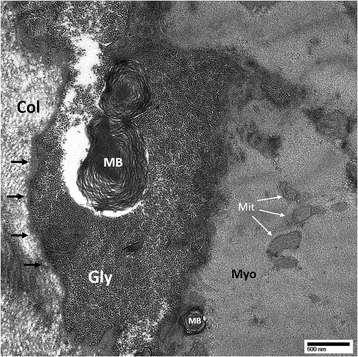
Transmission electron microscopy of the frozen endomyocardial biopsy tissue. There is marked cytoplasmic glycogen accumulation and occasional myelin bodies of various sizes. Gly = glycogen; MB = myelin body; Myo = myofibril; Mit = mitochondria; Col = extracellular collagen; black arrows denote cell membrane

Histology of the myocardial tissue revealed sub-sarcolemmal glycogen storage on Hematoxylin and Eosin (H&E) stain as well as Toluidine Blue stain (Fig. 1). The vast cytoplasmic glycogen accumulation was further confirmed on electron microscopy (Fig. 2). Glycogen storage in the tissue was also noted by a positive Periodic acid–Schiff (PAS) stain and digestion of glycogen with diastase. These findings further confirmed the clinical diagnosis.

## Discussion and conclusions

A genetic mutation linking familial HCM and WPW mapping to chromosome 7 band q3 was first described in 1995 [6]. This locus was later identified as the gene PRKAG2, which encodes for the y2 regulatory subunit of AMP-activated protein kinase (AMPK) [5]. AMPK is a heterotrimeric complex comprising a catalytic subunit (α) and two regulatory subunits (β and y) [7]. Activation of AMPK during acute low-energy states phosphorylates several downstream substrates and switches off ATP-consuming pathways such as glycogen, cholesterol and fatty acid synthesis. At the same time, AMPK activates ATP-producing pathways such as fatty acid oxidation, and it increases glucose uptake in cells [7, 8]. The mechanism by which PRKAG2 mutations lead to impaired glucose metabolism and excess glycogen storage in human cells is not well defined [9]. It has been proposed that early in the disease course the myocardial glucose uptake is elevated due to AMPK activation. However in advanced disease stages, AMPK may be suppressed by a negative feedback mechanism from the accumulated glycogen [9].

PRKAG2 syndrome leads to pronounced enlargement of myocytes with frequent intracellular vacuoles filled with glycogen. This is in contrast to sarcomeric gene mutations, which pathognomonically present with myofibrillar disarray on microscopy [4]. This distinct pathology is likely responsible for the unique clinical manifestations of the disease. Other case reports of Arg302Gln mutations show a similar pattern of asymmetric HCM and the presence of an accessory pathways in patients with a family history of HCM as well as patients with de novo mutations [10]. There are reports of patients with this mutation who present with cardiac arrest and require the implantation of a defibrillator [10]. However, no previous reports include the presence of coronary bridging, which we found on angiography.

PRKAG2 syndrome often manifests at a young adult age with a wide spectrum of presentations. A 12-year study of PRKAG2 syndrome in 45 mutations carriers from three families showed the mean initial age of presentation is 24. The most frequent initial presentations included palpitations (48%), syncope (28%), and pre-syncope (15%). However, the spectrum of presenting symptoms included chest pain, heart failure, myalgia, and epilepsy [11].

Evaluation of PRKAG2 patients by EKG frequently shows a short PR interval and delta waves. On echocardiogram, patients with PRKAG2 syndrome have been noted to have LVH with an eccentric distribution [11]. The natural history of PRKAG2 mutations reveals a slowly progressive, occasionally massive, increase in wall thickness in the majority of patients [11]. These findings are in contrast with sarcomere gene mutation forms of HCM, in which the myocardium undergoes a gradual decrease in hypertrophy with increasing age [12]. In addition, patients with sarcomeric gene mutations usually have an asymmetrical pattern of hypertrophy with predilection for the intraventricular septum [13]. With progression of the disease, PRKAG2 syndrome often evolves into a phase characterized by systolic dysfunction and left ventricular dilation. These features resemble dilated cardiomyopathy and can subsequently progress into heart failure [14].

PRKAG2 mutations have been linked to arrhythmias, particularly WPW syndrome. The underlying molecular mechanism for the arrhythmias is not entirely known, but it has been associated with the glycogen accumulation in the tissue. This association was elucidated in a study, which showed that inhibition of glycogen content accumulation in cardiomyocytes can effectively suppress arrhythmias in transgenic mice [14]. A different study suggested an alternative mechanism based on the observation that mutations in PRKAG2 lead to thinned and stretched annulus fibrosis, which is disrupted at the atrioventricular junction. These findings, along with the lack of identification of a histologically defined bypass tract, have suggested that the disruption in annulus fibrosis may be responsible for pre-excitation syndrome seen in PRKAG2 mutations [15].

It is important for clinicians to recognize various genetic syndromes and their unique clinical presentations in patients with suspected HCM. Patients presenting with features of HCM and conduction abnormalities may require further investigation into a unifying underlying genetic etiology for their disease presentation. This case highlights the utility of genetic testing in in diagnosing a patient with PRKAG2 syndrome, which was further confirmed by heart tissue biopsy. The CARE guidelines were followed in reporting this case.

## Abbreviations

AMPK: AMP-activated protein kinase
H&E: Hematoxylin and Eosin
HCM: Hypertrophic Cardiomyopathy
LAD: Left anterior descending artery
LVH: Left ventricular hypertrophy
NSTEMI: Non-ST Elevation Myocardial Infarction
PAS: Periodic acid–Schiff
PRKAG2: Protein Kinase AMP-Activated Non-Catalytic Subunit Gamma 2
WPW: Wolff-Parkinson-White
